# A Web-Based Communication Tool for Postoperative Follow-up and Pain Assessment at Home After Primary Knee Arthroplasty: Feasibility and Usability Study

**DOI:** 10.2196/34543

**Published:** 2022-04-28

**Authors:** Torbjørn Rian, Kari Sand, Eirik Skogvoll, Pål Klepstad, Tina S Wik

**Affiliations:** 1 Department of Anaesthesiology and Intensive Care Medicine St. Olavs Hospital Trondheim University Hospital Trondheim Norway; 2 Department of Neuromedicine and Movement Science Norwegian University of Science and Technology Trondheim Norway; 3 Department of Health Research SINTEF Digital Trondheim Norway; 4 Department of Circulation and Medical Imaging Faculty of Medicine and Health Sciences Norwegian University of Science and Technology Trondheim Norway; 5 Department of Orthopedic Surgery St. Olavs Hospital University Hospital of Trondheim Trondheim Norway

**Keywords:** feasibility studies, postoperative follow-up, primary knee arthroplasty, pain assessment, mobile application, pain treatment, follow-up at home

## Abstract

**Background:**

We report the use of an electronic tool, Eir (Eir Solutions AS, Norway), for symptom registration at home after knee arthroplasty. This electronic tool was used in a randomized controlled trial (RCT) comparing 3 different analgesic regimens with respect to postoperative pain and side effects.

**Objective:**

The aim of this substudy was to investigate this electronic tool for symptom registrations at home with respect to usability (ie, how easy it was to use) and feasibility (ie, how well the tool served its purpose).

**Methods:**

To assess the tool's usability, all participants were invited to fill out the 10-item System Usability Scale (SUS) after using the tool for 8 days. To assess feasibility, data regarding the participants' ability to use the tool with or without assistance or reminders were collected qualitatively on a daily basis during the study period.

**Results:**

A total of 134 patients completed the RCT. Data concerning feasibility of the web-based tool were collected from all 134 patients. The SUS was completed by 119 of the 134 patients; 70.2% (94/134) of the patients managed to use the tool at home without any technical support. All technical challenges were related to the login procedure or internet access. The mean SUS score was 89.6 (median 92.5; range 22.5-100).

**Conclusions:**

This study showed high feasibility and high usability of the Eir web tool. The received reports gave the necessary information needed for both research data and clinical follow-up.

**Trial Registration:**

ClinicalTrials.gov NCT02604446; https://www.clinicaltrials.gov/ct2/show/NCT02604446

## Introduction

### Background

Length of hospital stay (LOS) after hip and knee arthroplasty is shorter with modern fast-track surgery [1]. Therefore, symptoms and complications previously observed and treated in the hospital will now occur at home. These include risks for respiratory depression caused by opioid analgesics and infectious complications. Tools for active communication with the patients after early discharge from the hospital will be important to avoid or address these problems.

Benefits of communication via the internet using tablets and smartphones for postoperative follow-up after surgery have been reported by previous research [2-5]. Modern technology can provide detailed postoperative surveillance data at home [2], guide patients in postoperative pain management [3], provide detailed data on postoperative pain development and opioid use [4], and measure recovery of activity level after total joint arthroplasties [5]. Several advantages of using electronic tools for patient-recorded outcomes have been identified, such as fast and direct communication between patient and health personnel, higher data quality and response rates, easier storage of data, easier access to data for both patients and health personnel, access to real-time patient data for health care personnel, and easier connection of different sources of data [6]. Challenges when using electronic tools can be software failure when used at home or user errors, or the tool can be inconvenient and tiresome to use for the patients over time. These factors can lead to missing data [2,3,5]. Electronic tools should be user-friendly and understandable for the patients, and they should be able to obtain the information needed for clinical follow-up. Evaluations of electronic tools related to specific patient populations must highlight these features and should be done before implementing a tool in routine use.

### Objectives

We report the use of an electronic tool, Eir (Eir Solutions AS, Norway), for symptom registration at home after knee arthroplasty. This electronic tool was used in a randomized trial [7] comparing 3 different analgesic regimens with respect to postoperative pain and side effects. An electronic tool for symptom registration was initiated to closely monitor the patient’s course at home over the succeeding postoperative week. The aim of this substudy was to investigate this electronic tool for symptom registration at home with respect to usability (ie, how easy it was to use) and feasibility (ie, how well the tool served its purpose).

## Methods

### Study Design

The study was conducted and is reported according to the CONSORT-EHEALTH (Consolidated Standards of Reporting Trials of Electronic and Mobile Health Applications and Online Telehealth) checklist (Multimedia Appendix 1). In a randomized, double-blinded, placebo-controlled study comparing 3 different postoperative analgesic regimens [7], we used a web-based tool, Eir, for registration of patient-reported outcomes and medication [8,9] to evaluate the effect of the pain management and to assess side effects. The web-based tool was used by all patients regardless of intervention arm. We present the results for the full sample regardless of which intervention arm they were allocated to in the randomized controlled trial (RCT), as we did not expect the different postoperative analgesic regimens to influence the patients’ ability to use the tool. The patients registered pain levels, medication use, and side effects for the first 8 days after surgery. Two of the postoperative regimens included the use of opioids: oxycodone 10 mg twice daily or tapentadol 50 mg twice daily. All 3 groups had access to immediate-release oxycodone 5 mg as rescue medication for pain. Other sedative medications were not given as a part of the study interventions. Assessment of the tool's usability and feasibility was an integrated part of the trial. To assess the tool's usability, all participants were invited to fill out the 10-item System Usability Scale (SUS) after using the tool for 8 days. To assess feasibility, data regarding the participants' ability to use the tool with or without assistance or reminders were collected qualitatively on a daily basis during the study period.

### Patients and Setting

The study was a single-center, prospective, randomized, double-blinded trial carried out in a university hospital setting from November 26, 2015, to November 7, 2018. Patients scheduled for surgery with total knee arthroplasty (TKA) between 18 years and 80 years of age were considered for inclusion in the study. Exclusion criteria were cognitive impairment, inability to read or speak Norwegian, lack of a cell phone or wireless Wi-Fi connection at home, or use of drugs or medical conditions that conflicted with one or more of the study drugs or any of the multimodal basal pain medications given in the study. The trial was completed by 134 patients [7].

### The Web-Based Application

Eir is an electronic symptom assessment tool developed by the European Palliative Care Research Centre at the Norwegian University of Science and Technology and St. Olavs Hospital, Trondheim University Hospital for use in cancer care. A separate patient module was designed for patient-reported postoperative symptom assessment and medication registration after fast-track knee arthroplasty. It consisted of measurements of pain and side effects and detailed registration on use of analgesic drugs (Multimedia Appendix 2).

### Procedures

**Figure 1:**
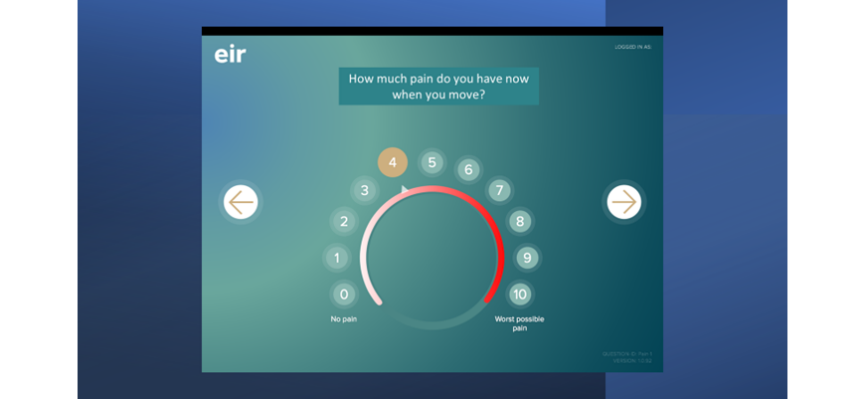
Picture of the application in use with English text (Norwegian text was used in the study).

Patient-reported data concerning effect and side effects of the pain treatment were registered daily for 8 days by use of Eir on a tablet and transferred wirelessly to the Eir database. The patients used Eir either on their personal tablet or on a tablet supplied by the study (Apple iPad Mini 2, 16 GB). The patients were introduced to the application and the tablet after surgery when awake and after mobilization. All patients were supervised for 2 electronic self-reports while hospitalized, to check that the system was working and that the patient was able to comply with the procedure. To be able to use Eir on tablets at home, the patient had to log on to a wireless Wi-Fi connection, use the correct password to log in, and answer 15 questions regarding use of study drug and other analgesics, pain intensity, and side effects of analgesics (Multimedia Appendix 2). Pain intensity and side effects were all measured on an 11-point numeric rating scale, from 0 to 10 (Figure 1).

All patients were instructed to self-report on the tablet each day before noon. A reminder was sent by the main author as an SMS if no registrations were received within the agreed time. The patient received a second reminder as a phone call if there were no registrations after the SMS reminder. The patient`s closest relative was contacted if none of these communication methods succeeded. All patients received a paper version of the tablet questions for back-up in case of technical failure.

### Data Collection Instruments

For each patient, the completeness of data, use of reminders, potential user problems, solution to problems, and phone calls were registered for feasibility assessment. Usability was measured by use of the 10-item SUS, which is designed to measure the subjective usability of websites and software [10,11]. The SUS gives a score ranging from 0 to 100, with higher scores meaning better usability. A paper version of the SUS questionnaire was given to the participants on the day of discharge from the hospital, and the participants were instructed to complete the form 8 days after surgery. All patients received a reminder about the SUS form as an SMS and returned the questionnaire by mail.

Two weeks after the operation, all patients were interviewed by telephone as a follow-up, and a global satisfaction score on the pain treatment was registered. The patients were given the opportunity to comment on any part of the treatment and follow-up after the operation.

The participants were grouped into 5 levels of technical skills, or tech groups. The 5 different groups were made by the first author (TR), based on the level of intervention needed to retrieve data (Table 1). The patients’ skill levels were compared with the registered data on side effects, drug consumption, age, and gender to assess if such factors could explain the difference in feasibility.

**Table 1:** Classification of the tech groups.

Tech group	Level of technical skills
1	No intervention was needed.
2	1-2 reminders were provided, but no technical support was needed.
3	Technical support was provided 1 time.
4	Technical support was provided several times.
5	Not able to use the application at all, all data were collected by paper forms.

### Statistical Methods

Descriptive statistics were reported as mean (SD) or median and 25th and 75th percentiles, as appropriate, according to the distribution of variables. The distribution of feasibility categories was reported as multinomial probabilities with exact 95% CIs. Correlation between feasibility and SUS and between feasibility and other clinical characteristics (eg, age, gender, type, and amount of drug received) was calculated using the Kendall nonparametric correlation coefficient and plotted appropriately. Correlation between feasibility and use of a personal tablet as opposed to those who borrowed a tablet from the hospital was calculated using the Mann-Whitney *U* test.

### Ethics

The study was approved by the Regional committee for Medical and Health Research Ethics (2015/209/Rek-Midt) and the Norwegian Medicines Agency (15/01581-13) and registered at clinicaltrials.gov (NCT02604446) on November 13, 2015. The study was conducted in compliance with the Declaration of Helsinki and Good Clinical Practice. Written informed consent was obtained from all participants before inclusion.

## Results

### Patients

A total of 134 patients (61 men and 73 women) between the ages of 32 years and 78 years completed the RCT. Data were collected from all 134 patients concerning feasibility of the web-based tool. The SUS was completed by 119 of the 134 patients.

### Evaluation Outcomes

The mean SUS score was 89.6 (median 92.5; range 22.5-100). This score corresponds to an A+ in a scoring system given by Sauro [12].

Most of the patients (94/134, 70.2%) managed to use Eir without any technical support (Table 2). Patient-reported data were provided by 68 patients for the defined period of 8 days without any intervention (tech group 1); 26 patients received 1 to 2 reminders but did not need any technical support (tech group 2); 24 patients received simple technical support once, typically on their first attempt to answer after hospital discharge (tech group 3); 10 patients received technical support several times (tech group 4); and 6 patients did not use the application system at home at all (tech group 5). For this tech group, all data after hospitalization were collected from the paper version.

In tech group 5, 1 patient was transferred to ward home for blood transfusion and never managed to connect to the internet, 1 patient could not find the password for his home network, and 1 patient was sent home without a tablet. These 3 patients did not complete the SUS. The last 3 patients in tech group 5 did not use the tablet at home and gave a SUS score based on their use of the application while hospitalized, their scores being 50, 62.5, and 85.

Of the 134 patients, 49 (37%) used their own tablet with the application installed, while 85 of 134 (63%) patients borrowed a study tablet in the registration period at home.

Patient demographics, study drug, level of self-reported side effects, and mean SUS score for each tech group related to their technical skill level is displayed in Table 2.

**Figure 2:**
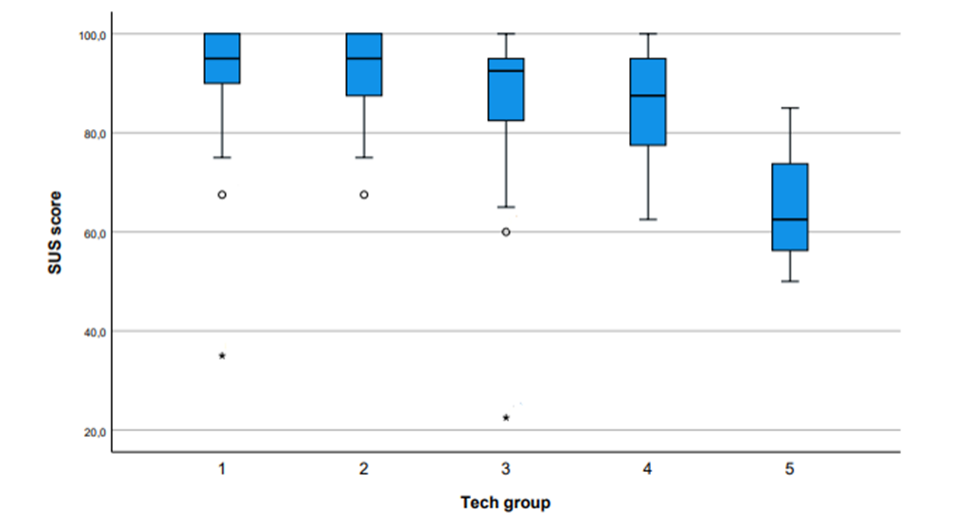
The relationship between tech group and System Usability Scale (SUS) score.

Figure 2 shows the relationship between tech group and SUS. We observed a significant negative correlation between technical performance (feasibility) and the patient´s evaluation of the electronic tool’s usability (SUS score; correlation coefficient –0.18; *P*=.02). The 6 patients who were not able to use or not interested in using the tablet for self-registration (tech group 5) had a significantly higher age than the rest of the patients (*P*=.004). We found a correlation between feasibility and use of personal tablet (*P*=.04) as opposed to those who borrowed a tablet from the hospital. We observed no gender differences related to technical skills (*P*=.16). There were no significant associations between the patients' technical skills and the use of study drug (placebo versus the opioid groups: tapentadol and oxycodone).

The total amount of reports possible was 1072 both at hospital and home. At home, the maximal total number of reports was 642, of which 631 (98.3%) was delivered. The patients delivered 450 of 631 reports (71.3%) at home without technical support.

**Table 2:** Patient demographics, study drug, level of self-reported side effects, and mean System Usability Scale (SUS) score for each tech group.

Characteristics		Tech group 1 (n=68): no intervention	Tech group 2 (n=26): 1-2 simple reminders	Tech group 3 (n=24): technical support once	Tech group 4 (n=10): support on multiple occasions	Tech group 5 (n=6): unable to use
Proportion of the total sample (95% CI)		0.51 (0.39-0.62)	0.19 (0.12-0.30)	0.18 (0.10-0.27)	0.07 (0.03-0.15)	0.05 (0.01-0.11)
**Sex, n (%)**						
	Male	29 (43)	10 (38)	12 (50)	6 (60)	4 (67)
	Female	39 (57)	16 (62)	12 (50)	4 (40)	2 (33)
Age (years), mean (SD)		61 (9.82)	58 (9.76)	64 (9.92)	62 (7.69)	71 (5.37)
LOS^a^ (days), mean (SD)		2.1 (0.66)	2.5 (0.76)	2.1 (0.58)	2.3 (0.95)	2.5 (0.84)
**Study drug in original trial, n (%)**						
	Oxycodone depot	23 (34)	12 (46)	6 (25)	3 (30)	2 (33)
	Tapentadol depot	24 (35)	8 (31)	10 (42)	3 (30)	0 (0)
	Placebo	21 (31)	6 (23)	8 (33)	4 (40)	4 (67)
SUS^b^ form completed, n (%)		61 (90)	25 (96)	21 (88)	9 (90)	3 (50)
SUS score, mean		91.8	92.9	85.2	84.4	65.8
Pain (at rest), mean^c^		2.17	2.35	2.09	3.26	1.21
Constipation, mean^c^		0.66	0.47	0.42	0.59	0.93
Dizziness, mean^c^		0.95	1.13	0.88	2.29	0.57
Headache, mean^c^		0.42	0.76	0.59	0.90	0.14
Nausea, mean^c^		0.88	1.08	0.99	1.79	0.76
Sedation, mean^c^		2.03	2.21	2.16	3.26	1.21
Sleep quality, mean^c^		2.97	3.4	3.49	4.53	1.98
Amount of rescue drug (oxycodone 5 mg tablet), mean value per 24 hours		1.59	2.3	1.79	3.2	1.02
Used personal tablet, n		30 (44)	9 (35)	7 (29)	2 (20)	1 (17)
Used borrowed tablet, n (%)		38 (56)	17 (65)	17 (71)	8 (80)	5 (83)

^a^LOS: length of hospital stay.

^b^SUS: System Usability Scale.

^c^Measured on a 0-10 numeric rating scale (0=best, 10=worst).

## Discussion

### Principal Findings

This study showed high feasibility and high usability of the Eir web tool. A large majority (94/134, 70.2%) of the patients managed to use the tool at home without any technical support. All technical challenges were related to the login procedure or internet access. The received reports gave the necessary information needed for both research data and clinical follow-up.

Almost 90% (119/134, 88.8%) of the patients completed the SUS to evaluate the usability of the electronic tool. Missing reports were evenly distributed between all technical skill groups. We found a clear correlation between the SUS score and feasibility of the tool (ie, the level of technical support given). In the tech groups with higher need of technical support, there was also a higher fraction of patients not having their own tablet, and those not able to complete the reports were older. This implies that the differences in feasibility and usability score between the tech groups are more related to technical experience and skills, rather than effects of the original study intervention or factors explained by the tool itself. The total score for usability was high and more than 80 in groups 1 to 4.

### Comparison With Prior Work

The electronic tool evaluated in this study has previously been tested among patient groups with cancer treated in hospital [8,9]. This is the first time the tool was evaluated for unassisted symptom registration at home. There are a few studies on electronic symptom assessment at home after surgery [2-5], indicating that electronic postoperative follow-up at home might be an emerging field. Previous studies for electronic follow-up have obtained different information. Chevallier et al [2] evaluated 29 patients using a home assessment tool after ambulatory surgery and measured pain, nausea, vomiting, comfort, oxygen saturation, heart rate, and blood pressure. Pombo et al [3] used an electronic tool for pain registration with 32 patients, which generated treatment recommendations for the patients after ambulatory surgery. Hajewski et al [4] used automated mobile messaging to gather detailed data on pain development and opioid utilization in 29 patients after periacetabular osteotomy. Lyman et al [5] used a smartphone application to measure step counts and patient-recorded outcome measures, including pain scores, with 267 patients after TKA and total hip arthroplasty (THA) surgery. These 4 studies had patients with mean ages of 47, 48, 22, and 61 years, respectively, while the mean age in our study was 61.5 years.

The technological tools and patient populations differ in these previous studies. The study by Chevallier et al [2] was a pilot study testing advanced monitoring equipment at home. The electronic tools provided 62% (2038/3248) of the expected data items compared with 82% (2656/3248) from a paper back-up. The major reason for missing data was software malfunction. The percentage of missing data in the study by Pombo et al [3] was 29.6%. They used an electronic pain diary based on periodic alarms to recommend pain treatment. The major reason for missing data was that the participant did not hear the alarm or it occurred at an inconvenient time. Hajewski et al [4] reported missing or incomplete data for 16% of the patients but stated no reason. In the study by Lyman et al [5], 6 months of follow-up was completed by 65% of THA patients and 68% of TKA patients. Reasons for noncompletion included time commitment, phone battery, app issues, and health complications. In our study, missing data were considerably less. We received 631 of 642 reports and hence, had only 1.7% (11/631) missing data. We used the tool to gather information in a drug study and had to assist 30% of the patients to avoid loss of data. The fraction of missing data is approximately the same in these studies, but with different causes. In our study, it was more important to gather all data regarding the effect of the different drugs than to let the patients use the electronic tool without assistance.

### Use in Research

The original trial [7] used this tool to obtain data for pain research. The pain data from the Eir database provide a detailed diary with daily pain scores with an exact time stamp and is a promising tool for pain research in the early postoperative period after early admission to home. Electronic assessment at home provides unbiased data from the patients and may be more reliable as the patients are not influenced by the investigators. As for other surgeries, the LOS after arthroplasty has been reduced in recent years, and in our study, the mean LOS was 2.1 days. This advocates for closer evaluation of symptoms at home.

### Limitations

There is no consensus in previous studies evaluating electronic tools on how to measure feasibility. The need for technical assistance has been defined differently between studies. Our intention was to describe the variation, and we found that a division into 5 tech groups was informative, as it elaborates the variation between the tech groups with regard to need for support. This symptom assessment system requires a well-developed technological infrastructure, which is present in Norway and many developed countries but not in all countries. It also demands a population that is familiar with use of technology. The study population is not necessarily representative of all patients operated with TKA since patients with cognitive impairment, an age older than 80 years, and inability to read or speak Norwegian were excluded from this study. For a home electronic registration of symptoms to be of use for patients, it must also be connected to an organization that monitors the patients’ responses and, if needed, intervenes.

### Conclusions

In this study, we evaluated an electronic symptom assessment tool that most patients used without technical support. The tool's usability was scored as high, and it seems as if the differences in feasibility and usability scores were more related to technical experience and skill rather than the tool itself or the clinical intervention in this study. An electronic tool that is easy to use for patients and does not require technical support can provide adequate follow-up for patients in the early postoperative period at home.

## Appendix

Multimedia Appendix 1 CONSORT-eHEALTH checklist (V 1.6.1).

Multimedia Appendix 2 Questions on tablet.

## Abbreviations

LOS: length of hospital stay
RCT: randomized controlled trial
SUS: System Usability Scale
THA: total hip arthroplasty
TKA: total knee arthroplasty
