# Supplementary material for: A Web-Based Communication Tool for Postoperative Follow-up and Pain Assessment at Home After Primary Knee Arthroplasty: Feasibility and Usability Study
Source: JMIR Form Res. 2022 Apr 28;6(4):e34543. doi: 10.2196/34543 (PMC9100538; doi:10.2196/34543)
Supplement: Multimedia Appendix 2 [file formative_v6i4e34543_app2.doc]

| Question | Alternatives |
| --- | --- |
| Have you taken your study drug as planned the last 24 hours? | Yes/No |
| If no, why not? | Forgot it/No pain/Too much side effects/Other |
| Have you taken the scheduled analgesics (4 doses of Paracetamol^a^ and two doses of Vimovo^b^) the last 24 hours? | Yes/No |
| If no, why not? | Forgot it/No pain/Too much side effects/Other |
| How many tablets of Oxynorm^c^ have you used the last 24 hours? | Number of 5mg tablets |
| You will now receive some questions regarding pain. Pain is rated on a 0 to 10 scale where 0 is no pain and 10 is the worst imaginable pain. |  |
| How much pain do you have now when you move? | NRS 0-10 |
| How much pain do you have now when you are at rest? | NRS 0-10 |
| How much pain have you had on average the last 24 hours when you move? | NRS 0-10 |
| How much pain have you had on average the last 24 hours at rest? | NRS 0-10 |
| How high was your highest pain score the last 24 hours? | NRS 0-10 |
| How much nausea have you had the last 24 hours? (0 is no nausea and 10 is the worst imaginable nausea) | NRS 0-10 |
| How much dizziness have you felt the last 24 hours? 0 is no dizziness and 10 is the worst imaginable dizziness) | NRS 0-10 |
| Have you passed stool the last 24 hours? | Yes/No |
| Do you feel constipated? (0 is no constipation and 10 is the worst imaginable constipation) | NRS 0-10 |
| Are you tired or sleepy? (0 is no sedation and 10 is the worst imaginable sedation) | NRS 0-10 |
| Have you had slept badly last night? (0 is the best imaginable sleep and 10 is the worst imaginable sleep) | NRS 0-10 |
| How much headache have you had on average the last 24 hours? (0 is no headache and 10 is the worst imaginable headache) | NRS 0-10 |

^a^Acetaminophen 1 g ^b^Naproxen 500 mg + esomeprazole 20 mg ^c^Oxycodone immediate-release 5 mg
